# Expression of Matrix Metalloproteinases and Their Tissue Inhibitors in Peripheral Blood Leukocytes and Plasma of Children with Nonalcoholic Fatty Liver Disease

**DOI:** 10.1155/2020/8327945

**Published:** 2020-09-10

**Authors:** Joanna B. Trojanek, Jacek Michałkiewicz, Renata Grzywa-Czuba, Wojciech Jańczyk, Lidia Gackowska, Izabela Kubiszewska, Anna Helmin-Basa, Aldona Wierzbicka-Rucińska, Mieczysław Szalecki, Piotr Socha

**Affiliations:** ^1^Department of Microbiology and Clinical Immunology, The Children's Memorial Health Institute, Warsaw, Poland; ^2^Collegium Medicum Nicolaus Copernicus University, Bydgoszcz, Poland; ^3^Department of Gastroenterology, Hepatology, Nutritional Disorders and Paediatric, The Children's Memorial Health Institute, Warsaw, Poland; ^4^Department of Biochemistry, Radioimmunology and Experimental Medicine, The Children's Memorial Health Institute, Warsaw, Poland; ^5^Department of Endocrinology and Diabetology, The Children's Memorial Health Institute, Warsaw, Poland; ^6^Department of Medicine and Health Sciences, Jan Kochanowski University, Kielce, Poland

## Abstract

Gene expression profiles of matrix metalloproteinases (*MMPs*) and their tissue inhibitors (*TIMPs*) were evaluated in peripheral blood leukocytes of children with nonalcoholic fatty liver disease (NAFLD). Gene expression patterns were correlated with their plasma protein counterparts, systemic parameters of liver injury, and selected markers of inflammation. The *MMP*-*2*, *MMP*-*9*, *MMP*-*12*, *MMP*-*14*, *TIMP*-*1*, *TIMP*-*2*, *TGF*-*β*, and *IL*-*6* transcripts levels were tested by the real-time PCR. Plasma concentrations of MMP-9, TIMP-1, MMP-9/TIMP-1 ratio, MMP-2/TIMP-2 ratio, sCD14, leptin, resistin, IL-1 beta, and IL-6 and serum markers of liver injury were estimated by ELISA. The *MMP*-*9*, *TIMP*-*2* expression levels, plasma amounts of MMP-9, TIMP-1, and the MMP-9/TIMP-1 ratio were increased in children with NAFLD. Concentrations of AST, ALT, GGT, and leptin were elevated in serum patients with NAFLD, while concentration of other inflammatory or liver injury markers was unchanged. The *MMP*-*2* and *MMP*-*9* levels correlated with serum liver injury parameters (ALT and GGT concentrations, respectively); there were no other correlations between *MMP/TIMP* gene expression profiles, their plasma counterparts, and serum inflammatory markers. Association of *MMP*-*2* and *MMP*-9 expression with serum liver injury parameters (ALT, GGT) may suggest leukocyte engagement in the early stages of NAFLD development which possibly precedes subsequent systemic inflammatory responses.

## 1. Introduction

Nonalcoholic fatty liver disease (NAFLD) is a chronic inflammatory disorder, closely related to metabolic syndrome components, like obesity and type 2 diabetes mellitus. The disease is defined as liver fat accumulation (steatosis) that exceeds 5% of hepatocytes and is not secondary to genetic and metabolic disorders, infections, alcohol consumptions, or malnutrition [1]. NAFLD refers to the spectrum ranging from a simple steatosis to nonalcoholic steatohepatitis (NASH), fibrosis, and cirrhosis, with possible progression to hepatocellular carcinoma. Those histopathological changes not only occur within the hepatocytes [2] but primarily focus on active remodeling of the liver extracellular matrix (ECM) [3]. This process leads to excessive deposition of ECM compounds, such as type I, III, and IV collagens, and enhances expression of noncollagenous components including fibronectins, laminins, proteoglycans, and elastins [4]. Considering that all ECM compounds are degraded by endopeptidases called matrix metalloproteinases (MMPs), and whose activities are controlled by their endogenous tissue inhibitors (TIMPs), MMPs and equally TIMPs become the main players in rising fibrotic changes in the course of NAFLD progression [5, 6].

MMPs are involved in degradation and remodeling of ECM protein in both the physiological and pathological conditions. Natural, physiological MMP inhibitors (TIMPs) regulate the proteolytic MMP activity in tissues, forming with them stable noncovalent bonds. According to this, a healthy liver has a moderate ECM turnover that correlates with a small amount of metalloproteinases, as well as TIMPs constitutively expressed in normal livers [7], while any disruption of MMP activity and imbalance in the expression of MMP/TIMP system components [5] often leads to tissue damage and functional alternation triggering pathological conditions like liver fibrosis.

Liver MMP/TIMP components originate from stellate cells, sinusoidal endothelial cells (LECs), residual macrophages (Kupffer cells), and a variety of other cell types migrating to the liver from peripheral tissues including monocytes, neutrophils, and lymphocytes [8], as well as bone marrow-derived precursors [9]. It is worth noticing that leukocyte expression of the MMP/TIMP system plays an important role in the development of local inflammation, its resolution, and tissue repair.

Most types of leukocytes [10–13] have been shown to be engaged in liver injury and repair. Liver infiltration by inflammatory leukocytes is controlled by chemokines, which determine the composition and activation status of immune cells recruited to the injured liver [14, 15]. Therefore, we assumed that peripheral blood leukocytes in children with NAFLD will respond to liver injury through changes in their *MMP/TIMP* expression patterns, as well as *IL*-*6* and *TGF*-*β* gene expression profiles, and that these changes will more or less correspond to plasma inflammatory markers related to liver injury.

Leukocyte expression of MMP-2, MMP-9 (gelatinases), and MMP-14 (a membrane-type MMP) enhances their abilities for liver infiltration during inflammatory responses (basement membrane degradation) where they participate in ECM remodeling and regulation of inflammatory responses by modifying chemokine and cytokine activities [16]. The MMP-2, MMP-9, and MMP-14 degrade denatured collagen (gelatin) and other collagen types, as well as noncollagenous ECM components such as elastin, fibronectin, and laminin [17]. Macrophage MMP-12 is characterized by especially strong antielastin activity and plays an important role during liver fibrosis and fibrosis resolution [18]. Additionally, TIMP-1 preferentially complexes with pro-MMP-9, while TIMP-2 interacts with pro-MMP-2 [19]. The purpose of this study was to assess whether the profile of the MMP/TIMP system expression in peripheral leukocytes and plasma samples of children with NAFLD may reflect the disease activity or serve as an early marker of an ongoing disease process that eventually precedes the appearance of clinical symptoms of NAFLD.

## 2. Materials and Methods

### 2.1. Study Subjects

This study has been conducted according to the Declaration of Helsinki and with the approval from the Children's Memorial Health Institute Ethics Committee. All patients and parents gave informed consent to participate in the study. A total of 35 patients with NAFLD (aged 14.2 ± 2.6 years; BMI 29.3 ± 4.7) and 37 healthy lean control volunteers (aged 14.7 ± 2.6; BMI 21.2 ± 3.7) were included in the study. The exclusion criteria were as follows: renal insufficiency, viral and autoimmune liver damage, storage disease, hepatic cirrhosis, type 1 and 2 diabetes, chronic diseases of the gastrointestinal tract, taking lipid-lowering or lipid metabolism-affecting drugs. Diagnostic criteria were (a) ultrasound findings of fatty liver disease [20] and (b) increased ALT activity. The upper limit of the normal ALT value established for healthy children was 25.8 U/L (boys) and 22.1 U/L (girls) [21]. In all patients and healthy adolescents, the general clinical examination was conducted including anthropometry with BMI calculation and waist and hip measurement with *W*/*H* ratio counting. Venous blood was collected from an antecubital vein after at least 12 h of fasting. This blood was used to measure serum levels of total cholesterol (TC), triglycerides (TG), low-density lipoprotein-cholesterol (LDL-C), high-density lipoprotein-cholesterol (HDL-C), *γ*-glutamyltransferase (GGT), alanine aminotransferase (ALT), aspartate aminotransferase (AST), and high-sensitivity C-reactive protein (hs-CRP) levels by enzymatic commercial biochemical tests (Roche Diagnostics; Risch-Rotkreuz, Switzerland). The hexokinase method was used to measure glucose and immunoradiometric assays (Biosources; San Diego, CA, USA) for insulin concentration. Insulin resistance was calculated as follows: (fasting glucose × fasting insulin)/22.5, according to the homeostasis model assessment of insulin resistance (HOMA-IR) [22] (Table 1).

### 2.2. RNA Isolation and Real-Time PCR Technique

Details concerning the PCR technique used here were previously described [23]. Briefly, peripheral blood leukocytes were obtained by Histopaque (Sigma-Aldrich 1119; Saint Louis, MO, USA) gradient centrifugation. Total RNA was isolated with the Chomczyński method using TRIzol Reagent (Ambion; Carlsbad, CA, USA). By using absorbance at 260 nm and 280 nm, each sample RNA concentration was determined and purity/integrity was checked. One microgram of total RNA per sample was converted into cDNA via Reverse Transcription Polymerase Chain Reaction (RT-PCR) by using TaqMan Reverse Transcription Reagents. Quantitative RT-PCR (real-time PCR) was used for the following target genes: *MMP*-*9*, *MMP*-*2*, *MMP*-*12*, *MMP*-*14*, *TIMP*-*1*, *TIMP*-*2*, *TGF*-*β*, *IL*-*6*, and the endogenous control (reference gene) glyceraldehyde-3-phosphate dehydrogenase (*G3PDH*). The tests were performed using the ViiA 7 Real-Time System, according to the manufacturer's recommendation. For one reaction, 50 ng of cDNA was applied with SYBR Green PCR Master Mix and 10 nmol/L for each of the forward and reverse primers (Table 2). The sample was run twice, and the average was taken to analysis. The specificity of the amplification reaction was verified by analysis of the melting curve. Relative fold changes in target gene expression between the NAFLD patients and the control group were determined by normalization of expression of the reference gene *G3PDH*, by using Pfaffl's mathematical model [24]. All reagents, equipment, and other supplies used for the real-time PCR technique were provided by Applied Biosystems distributed by Thermo Fisher Scientific Inc. (Waltham, MA, USA).

### 2.3. ELISA Method

Peripheral blood plasma was collected using EDTA as an anticoagulant. The DuoSet ELISA kits (R&D Systems; Minneapolis, MN, USA) were used to determine the total concentration of MMP-9 and TIMP-1, as well as MMP-9/TIMP-1 ratio, MMP-2/TIMP-2 ratio, and sCD14. Leptin and resistin levels were determined with the use of ELISA kits (DRG International (Springfield, NJ, USA) and Phoenix Pharmaceuticals (Burlingame, CA, USA), respectively), and plasma amounts of IL-1 beta and IL-6 were determined by the use of OptEIA Set ELISA kits (Becton Dickinson; Franklin Lake, NJ, USA) as previously described [25].

### 2.4. Statistical Analysis

The data were analyzed using the Statistica 13.1 software package. The data distribution pattern was tested by using the Shapiro-Wilk *W* test, and the appropriate statistical tests were chosen: parametric (Student's *t*-test) or nonparametric (Mann-Whitney *U* test). All data were not normally distributed. The differences between the two groups (patients vs. control) were estimated by using the nonparametric tests. The relation between the clinical, anthropometric, biochemical, molecular, and immunological parameters was assessed by Spearman's rank correlation analysis. The differences and correlation indexes were considered significant at *p* < 0.05.

## 3. Results and Discussion

**Figure 1:**
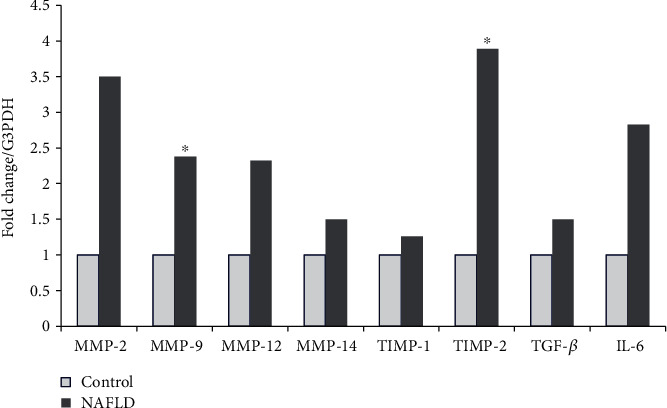
Gene expression in children with NAFLD. Transcript levels of *MMP*-*9*, *TIMP*-*1*, *MMP*-*2*, *TIMP*-*2*, *MMP*-*12*, *MMP*-*14*, *TGF*-*β*, and *IL*-*6* in leukocytes from the control group (open bars) and children with NAFLD (solid bars). Data are expressed as fold changes from children with NAFLD versus the control (*n* = 35/37). ^∗^Statistically significant at <0.01 by the Mann-Whitney *U* test.

We showed that the levels of leukocyte mRNA expression of *MMP*-*9* and *TIMP*-*2* but not *TIMP*-*1* were significantly higher in patients with NAFLD than in their healthy counterparts (2.38 (*p* = 0.001) and 3.89 (*p* = 0.002) fold change, respectively) (Figure 1).

**Figure 2:**
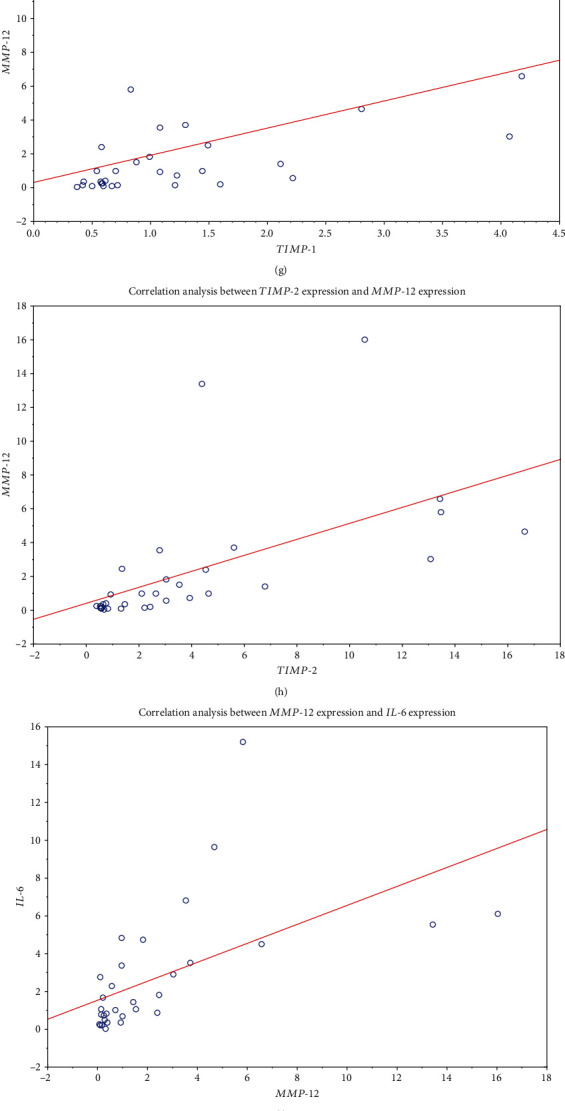
Spearman's correlation analysis between the expression of metalloproteinases, tissue inhibitors, their plasma protein counterparts, and liver injury markers: *MMP*-*9* and *TIMP*-*1* (a); *MMP*-*9* and *TIMP*-*2* (b); *MMP*-*9* and *MMP*-*12* (c); *MMP*-*2* and *TIMP*-*1* (d); *MMP*-*2* and *TIMP*-*2* (e); *TIMP*-*1* and *TIMP*-*2* (f); *TIMP*-*1* and *MMP*-*12* (g); *TIMP*-*2* and *MMP*-*12* (h); *MMP*-*12* and *IL*-*6* (i); *MMP*-*9* and GGT (j); *MMP*-*2* and ALT (k); MMP-9 and TIMP-1 (l); MMP-9 and MMP-9/TIMP-1 ratio (m); ALT and ASP (n); ALT and GGT (o).

*MMP*-*9* expression correlated inversely with *TIMP*-*1* (*r* = −0.587, *p* < 0.05), *TIMP*-*2* (*r* = −0.470, *p* < 0.05), and *MMP*-*12* (*r* = −0.573, *p* < 0.05) and positively with GGT serum concentration (*r* = 0.432, *p* < 0.05). Leukocyte *MMP*-*2* expression correlated positively with *TIMP*-*1* (*r* = 0.404, *p* < 0.05), *TIMP*-*2* (*r* = 0.375, *p* < 0.05), and serum ALT levels (*r* = 0.344, *p* < 0.05). Additionally, *MMP*-*12* expression correlated positively with *TIMP*-*1* (*r* = 0.598, *p* < 0.05), *TIMP*-*2* (*r* = 0.811, *p* < 0.05), and *IL*-*6* (*r* = 0.753, *p* < 0.05). Finally, *TIMP*-*1* levels of expression correlated with *TIMP*-*2* (*r* = 0.713, *p* < 0.05) (Figures 2(a)–2(k)).

MMP-9 and TIMP-1 plasma concentrations, as well as the plasma MMP-9/TIMP-1 ratio and leptin levels, were significantly increased in the patients with NAFLD as compared to the control group (Table 3).

The elevated MMP-9 plasma levels were positively correlated with TIMP-1 (*r* = 0.533, *p* < 0.05) and MMP-9/TIMP-1 ratio concentration (*r* = 0.848, *p* < 0.05). Moreover, ALT was correlated with concentrations of AST (*r* = 0.848, *p* < 0.05) and GGT (*r* = 0.494, *p* < 0.05) (Figures 2(l)–2(o)).

We found that the children with NAFLD were characterized by increased leukocyte *MMP*-*9* and *TIMP*-*2* expression, negative correlation of leukocyte *MMP*-*9* with *TIMP*-*1* and *TIMP*-*2*, and elevated plasma levels of MMP-9, TIMP-1, and plasma MMP-9/TIMP-1 ratio. The leukocyte *MMP*-*2* expression correlated with ALT serum levels, and the leukocyte *MMP*-*9* expression correlated with GGT serum concentrations. There were no other associations between leukocyte *MMP/TIMP* gene expression levels, their plasma protein counterparts, and serum markers of liver injury (ALT, AST, and GGT) or systemic inflammation. Leukocyte *MMP*-*9* upregulation may precede further stages of NAFLD development and subsequent systemic inflammatory responses reflected here by elevated plasma levels of total MMP-9, TIMP-1, and MMP-9/TIMP-1 ratio. However, the engagement of other proinflammatory immune response components remains limited (as demonstrated here by the unchanged plasma levels of IL-1 beta and IL-6).

Increased leukocyte *MMP*-*9* levels, unchanged *TIMP*-*1* expression, and positive correlation of *MMP*-9 expression with plasma GGT and *MMP*-*2* expression with serum ALT concentrations indicate the role of MMP-9 and MMP-2 gelatinases in NAFLD progression. MMP-9 is an inducible gelatinase expressed by all leukocyte types and other tissues including the native liver, albeit at very low levels [26]. In inflammatory conditions such as NAFLD, MMP-9 may play a role as an important mediator of leukocyte-induced liver damage [27, 28].

ECM degrading potential depends on the balance between TIMPs and MMPs [29]. Unchanged leukocyte *TIMP*-*1* levels with high *MMP*-*9* expression and elevated plasma MMP-9/TIMP-1 ratio may indicate increased MMP-9 activity (here high concentration of the MMP-9/TIMP-1 ratio) that should subsequently result in clearance of the fibrotic matrix. In contrast, TIMP-1 overexpression inhibits fibrotic matrix degradation and leads to extensive accumulation of interstitial ECM [30, 31]. Normal serum MMP-2/TIMP-2 ratio levels along with increased MMP-9/TIMP-1 concentrations may suggest rather low profibrotic MMP-2 activities at this stage of the disease. It has previously been found that leukocyte *MMP*-*9* and MMP-9 plasma expression levels correlated with increased IL-6 and IL-1 beta production. In contrast, both *MMP*-*2* and MMP-2 levels were associated with the production of anti-inflammatory cytokines (IL-4, IL-10) with profibrotic potential [32] indirectly confirming the role of MMP-2 in the development of liver fibrosis. These data may suggest that leukocyte MMP-9 and MMP-2 levels can mutually control each other to balance the inflammatory and anti-inflammatory activities necessary to regulate functions of the liver. Here, we found a positive correlation between leukocyte *MMP*-*2* expression and plasma ALT levels, confirming the role of leukocyte *MMP*-*2* in liver damage [33]. As previously reported, MMP-2 is strongly engaged in liver ECM remodeling and fibrosis [34]. We found that the levels of the plasma MMP-2/TIMP-2 ratio remained unchanged in patients with NAFLD in comparison to the control group. It may suggest that in the early stage of NAFLD (child model), the liver's profibrotic response to injury was rather low because the increase in MMP-2 plasma levels has previously been considered a sensitive marker of fibrosis in the early stage of NASH [35]. As described here, the positive correlations between leukocyte *MMP*-*12* expression and leukocyte *IL*-*6*, *TIMP*-*1*, and *TIMP*-*2* levels suggest that IL-6-induced MMP-12 expression may be counterbalanced by TIMPs. MMP-12 plays a key role in elastin degradation, so this observation may suggest that peripheral monocytes in children with NAFLD have decreased MMP-12 activities that result in diminution of their capacity for fibrosis resolution [36]. On the other hand, the expression of leukocyte *TGF*-*β* (strong fibrosis inducer) remained unchanged, suggesting rather low ability for its profibrotic action in the early stage of NAFLD [37].

## 4. Conclusions

Altogether, our data suggest the following: (a) changes in leukocyte *MMP/TIMP* expression profiles are mostly unrelated to their plasma levels but may represent early markers of leukocyte subset activation that eventually may precede the subsequent liver response to damage in the early stage of NAFLD, and (b) the subsequent increase in plasma levels of MMP-9 and TIMP-1 as well as the elevation of MMP-9/TIMP-1 ratios may reflect NAFLD's progress towards fibrosis, which is in agreement with the recent data on the role of TIMPs (especially TIMP-1) in the regulation of the matrix turnover [38].

## Figures and Tables

**Table 1:** Anthropometric and clinical characteristics of the study (NAFLD) and control groups.

	NAFLD	Control
No.	35	37
Age (years)	14.2 ± 2.6	14.7 ± 2.6
Height (cm)	164.5 ± 15	171.7 ± 17
Weight (kg)	80.3 ± 20	63.0 ± 16.5
BMI (kg/m^2^)	29.3 ± 4.7	21.0 ± 3.0
SDS-BMI	2.2 ± 0.9	0.2 ± 0.8
ALT (IU/L)	66.7 ± 44.8	16.2 ± 7.2
AST (IU/L)	40.0 ± 20.9	19.6 ± 6.0
GGT (IU/L)	45 ± 32	21.6 ± 4.2
TG (mg/dL)	108 ± 52	84 ± 44
TC (mg/dL)	187 ± 53	163 ± 34
HDL-C (mg/dL)	43.7 ± 13	46.3 ± 14
LDL-C (mg/dL)	123 ± 46	101 ± 34
hs-CRP (mg/dL)	0.31 ± 0.2	0.17 ± 0.1
Waist circumference	97.3 ± 12.7	73.9 ± 10
Hip circumference	101 ± 12	92.4 ± 10
WHR	0.96	0.82
Fasting glucose (mg/dL)	82.5 ± 9.8	86.0 ± 6.8
Fasting insulin (*μ*IU/mL)	15.5 ± 8.1	13.9 ± 5.9
HOMA-IR	3.2 ± 1.8	3.2 ± 1.5

ALT: alanine aminotransferase; AST: aspartate aminotransferase; GGT: gamma-glutamyltransferase; TG: triglycerides; TC: total cholesterol; HDL-C: high-density lipoprotein-cholesterol; LDL-C: low-density lipoprotein-cholesterol; hs-CRP: high-sensitivity C-reactive protein; WHR: waist to hip ratio; HOMA-IR: homeostasis model assessment of insulin resistance.

**Table 2:** Primer sequence of target genes and reference gene for SYBR Green real-time PCR.

Gene	Forward primer	Reverse primer
*MMP*-*9*	CAA CAT CAC CTA TTG GAT CC	CGG GTG TAG AGT CTC TCG CT
*MMP*-*2*	TGA TCT TGA CCA GAA TAC CAT CGA	GGC TTG CGA GGG AAG AAG TT
*TIMP*-*1*	CTT CTG GCA TCC TGT TGT TG	AGA AGG CCG TCT GTG GGT
*TIMP*-*2*	CGA CAT TTA TGG CAA CCC TAT CA	CAG GCC CTT TGA ACA TCT TTA TCT
*MMP*-*12*	TTCCCCTGAACAGCTCTACAAGCCTGGAAA	GATCCAGGTCCAAAAGCATGGGCTAGGATT
*MMP*-*14*	CGC TAC GCC ATC CAG GGT CTC AAA	CGC TAC GCC ATC CAG GGT CTC AAA
*TGF*-*β*	GGA AAC CCA CAA CGA AAT CTA TG	CGG GTT CAG GTA CCG CTT C
*IL*-*6*	TGA AAG CAG CAA AGA GGC ACT	GGC AAG TCT CCT CAT TGA ATC C
*G3PDH*	GCG GGG CTC TCC AGA ACA TCA T	CCA GCC CCA GCG TCA AAG GTG

*MMP*-*2*, *9*, *12*, and *14* indicate matrix metalloproteinase-2, 9, 12, or 14, respectively. *TIMP*-*1* and *2* indicate tissue inhibitor of metalloproteinase-1 or 2. *TGF*-*β*: transforming growth factor beta; *IL*-*6*: interleukin-6; *G3PDH*: glyceraldehyde-3-phosphate dehydrogenase.

**Table 3:** Plasma levels of metalloproteinases and their tissue inhibitors as well as cytokines and adipokines in the children with NAFLD and control groups.

Children group	sCD14 (ng/mL)	MMP-9 (ng/mL)	TIMP-1 (ng/mL)	MMP-2/TIMP-2 (ng/mL)	MMP-9/TIMP-1 (ng/mL)	IL-1 beta (pg/mL)	IL-6 (pg/mL)	Leptin (ng/mL)	Resistin (ng/mL)
NAFLD group size (*n* = 35)	1316 ± 145	63 ± 30^∗^	194 ± 137^∗^	133 ± 79	9.6 ± 7.7^∗^	5.6 ± 5.3	7.7 ± 7.0	17.3 ± 14.4^∗^	6.2 ± 4.5
Control group size (*n* = 37)	1216 ± 340	25 ± 19	87 ± 44	136 ± 44	2.2 ± 1.7	5.8 ± 2.8	5.7 ± 4.7	2.0 ± 1.5	3.9 ± 1.2

^∗^Statistically significant at <0.01 by the Mann-Whitney *U* test.

## Data Availability

All data generated or analyzed during this study are included in this published article.
